# Prevalence of vitamin E inadequacy, dietary intake and sources of alpha-tocopherol, and predictors of alpha- and gamma-tocopherol status in adolescent girls in Central Mozambique

**DOI:** 10.1017/jns.2023.103

**Published:** 2023-12-01

**Authors:** Liisa Korkalo, Georg Alfthan, Lourdes Fidalgo, Riitta I. Freese

**Affiliations:** 1 Department of Food and Nutrition, University of Helsinki, Helsinki, Finland; 2 Finnish Institute for Health and Welfare, Helsinki, Finland; 3 Food Security and Nutrition Association (ANSA), Maputo, Mozambique

**Keywords:** Breastfeeding, Food consumption, Sub-Saharan Africa, Reproductive age, Teenage, Vitamin E

## Abstract

An adequate alpha-tocopherol status is important for females at reproductive age. We studied the dietary intake and sources of alpha-tocopherol and alpha- and gamma-tocopherol status indicators in 14–19-year-old girls in Central Mozambique. We also explored factors associated with alpha- and gamma-tocopherol status. The participants (*n* 508) were from the cross-sectional ZANE Study that was conducted in 2010. We recruited two separate samples, one in January–February and the other in May–June. We collected venous blood samples and conducted 24 h dietary recall interviews. At the time of blood sampling, 11 % of participants were pregnant and 10 % were lactating. In the total sample, both seasons combined, the median intake of alpha-tocopherol was 6⋅7 mg/d, the mean plasma alpha- and gamma-tocopherol concentrations were 13⋅5 and 0⋅75 μmol/l, respectively, and the prevalence of vitamin E inadequacy (alpha-tocopherol <12 μmol/l) was 36⋅7 % (95 % CI: 31⋅9–42⋅0 %). Season and lactation status were significant predictors of alpha-tocopherol status regardless of which the three indicators (plasma concentration, alpha-tocopherol:total cholesterol ratio, gamma-tocopherol:alpha-tocopherol ratio) were used. Being a lactating mother was negatively associated and having a blood sample taken in January–February, when the main sources of alpha-tocopherol were mango and dark green leafy vegetables, was positively associated with alpha-tocopherol status. In conclusion, vitamin E inadequacy was common in Central Mozambique, and the status may fluctuate due to seasonal changes in the diet. We suggest that lactating mothers are specifically at risk of poor alpha-tocopherol status in resource-poor settings.

## Introduction

Vitamin E, more specifically alpha-tocopherol, is a lipid-soluble antioxidant that prevents oxidative damage to membrane lipids by scavenging free radicals. It is also involved in other functions such as immune response.^(1)^ Alpha-tocopherol has specific roles in pregnancy. Experimental studies have shown that alpha-tocopherol plays a critical role in neuro-embryogenesis.^(2)^ A large study examining the risk of miscarriage in pregnant women in rural Bangladesh supported the importance of good alpha-tocopherol status in early pregnancy. With regard to gamma-tocopherol, the results suggest that higher concentrations may be disadvantageous in themselves or indicative of a less beneficial diet and other environmental exposures. The researchers found that a lower plasma alpha-tocopherol concentration was associated with an increased risk of miscarriage, whereas a lower plasma gamma-tocopherol status was associated with a decreased risk of miscarriage.^(3)^ Furthermore, a systematic review found some, although inconsistent, evidence that alpha-tocopherol concentrations are lower in pregnant women who develop pre-eclampsia.^(4)^ The review authors noted that the current evidence is not, however, sufficient to rule out reverse causality (i.e. that the lower alpha-tocopherol concentrations could be a consequence, rather than a cause of pre-eclampsia) and that further studies are needed to elucidate this relationship.

The Institute of Medicine has set the estimated average requirement (EAR) of alpha-tocopherol at 12 mg/d for non-pregnant and pregnant females from the age of 14 years upwards and at 16 mg/d for lactating females.^(5)^ The respective recommended dietary allowances were set at 15 and 19 mg/d. The European Food Safety Authority (EFSA), on the other hand, has considered that there is insufficient evidence to set an EAR and has set an adequate intake value at 11 mg/d for females from the age of 10 years upwards, regardless of pregnancy and lactation status.^(6)^ In affluent countries, vegetable oils and other added fats are typically the most important sources of dietary alpha-tocopherol, but several other food groups, such as nuts and seeds, cereals, fish, and some fruit and vegetables, are also relevant sources.^(7,8)^ Less is known about the contribution of different sources in Sub-Saharan Africa.

There is no clear consensus on the cut-offs for plasma/serum alpha-tocopherol concentrations to determine the prevalence of vitamin E deficiency in different population groups. However, 12 μmol/l is commonly used as a cut-off, and, according to Traber,^(9)^ it indicates vitamin E insufficiency, if not frank deficiency. Mean plasma/serum concentrations above 20 μmol/l have been commonly reported in studies in Europe and North America.^(10,11)^ Studies in Sub-Saharan African countries have reported mean/median plasma/serum concentrations from 9⋅4 to 16⋅1 μmol/l for infants, children, and adolescents,^(12–17)^ from 4⋅8 to 19⋅1 μmol/l for non-pregnant adults and elderly,^(15,18–23)^ and from 11⋅0 to 25⋅5 μmol/l for women who were pregnant or up to 2 weeks post-partum.^(17,20,24–27)^

Alpha-tocopherol is transported in the blood in lipoproteins and since blood cholesterol levels vary in different populations and age groups, the alpha-tocopherol:total cholesterol ratio is also of interest as an indicator of alpha-tocopherol status. On the other hand, lipoproteins may be low in cases of protein–energy undernutrition; therefore, undernutrition may mask vitamin E deficiency.^(9)^ A third indicator that has been suggested for the interpretation of vitamin E status is the ratio of plasma gamma-tocopherol to alpha-tocopherol.^(28)^ It has been shown that when alpha-tocopherol intake increases, plasma gamma-tocopherol concentrations decrease.^(29)^ This can be explained by experimental work, demonstrating that when alpha- and gamma-tocopherols are simultaneously given to subjects in equimolar amounts, gamma-tocopherol is preferentially and rapidly metabolized and excreted from the body, whereas alpha-tocopherol is maintained in the circulation.^(30)^ Data on gamma-tocopherol concentrations, tocopherol:total cholesterol ratios, or gamma-tocopherol:alpha-tocopherol ratios in Sub-Saharan African populations are, however, scarce.

Seasonal fluctuations in plasma alpha-tocopherol concentrations have been observed in a study in Gambia,^(20)^ but overall, little is known about how season or socio-demographic factors, such as rural–urban residence, are related to alpha-tocopherol intake and plasma concentrations of different tocopherols in Sub-Saharan African countries. To our knowledge, there are no previous reports based on dietary assessment on how different food groups contribute to the total dietary intake of alpha-tocopherol or how dietary sources vary by season in Sub-Saharan African populations. Shedding light on tocopherol status among females of child-bearing age, especially in resource-poor settings, where they are vulnerable to undernutrition, is thus important. Our aim here was to describe the dietary intake and sources of alpha-tocopherol, the plasma concentrations of alpha- and gamma-tocopherols, and the prevalence of vitamin E insufficiency among adolescent girls in Mozambique, a country with a high rate of adolescent pregnancies.^(31)^ Additionally, we explored factors associated with alpha- and gamma-tocopherol status in this group.

## Methods

### Sample

The ZANE Study (Estudo do Estado Nutricional e da Dieta em Raparigas Adolescentes na Zambézia) was a population-based cross-sectional study that was conducted in 2010. A detailed description of sampling design, recruitment, and fieldwork methods is available in open access format.^(32)^ Briefly, two separate samples of adolescent girls were recruited in Zambézia Province, Central Mozambique. The first sample was recruited and studied in January–February, the ‘hunger season’, and the second in May–June, the harvest season. The sampling was carried out in five areas: (1) Quelimane, (2) the district town of Maganja da Costa, (3) rural villages in Maganja da Costa, (4) the district town of Morrumbala, and (5) rural villages in Morrumbala. Here, Quelimane is referred to as an urban area, and the other areas as rural. Girls were recruited from 40 different localities (neighbourhoods and villages) within these areas. Within each locality, local recruiters moved from house to house to recruit adolescent girls. The recruitment procedure was repeated in May–June in the same localities. Each participant signed an informed consent form. If she was under 18 years of age, a parent, husband, or guardian also signed the form. If the girl or the adult was illiterate, a fingerprint or a signature of a witness indicated informed consent. Age was verified by checking identity cards whenever possible. Alternatively, the time of birth was estimated with the help of family members.

Two health centres in Quelimane and two district hospitals served as study centres where measurements were taken and interviews were conducted. The consenting girls were invited to come to the study centre and participants who lived far from the study centre were provided with transport by car. A total of 551 girls were studied. For the present paper, we included all participants for whom plasma tocopherol and total blood cholesterol concentrations were available (*n* 508, 92 %). The participants were aged 14–19 years. The study was approved by the Bioethical Committee of the Ministry of Health in Mozambique. The study was registered at ClinicalTrials.gov (NCT01944891).

### Interviews and anthropometry

A single 24 h dietary recall interview was conducted for 501 (99 %) of the participants included in this paper. A sub-sample of these participants was invited to three additional 24 h dietary recall interviews. The second interview was successfully completed for 104 girls (in January–February). The third interview was completed for ninety-two girls (in May–June) and the third one for eighty girls (also in May–June). The first interview took place at the study centre, and the three subsequent interviews took place at participants’ homes.

During each interview, the participant was asked to report all food and beverages consumed the previous day. A set of food photographs and household utensils were shown to the participant to help her with the estimation of portion sizes. To calculate nutrient intake, we compiled a Mozambican food composition database.^(33)^ A large part of the vitamin E values (as alpha-tocopherol) were taken from the United States Department of Agriculture National Nutrient Database for Standard Reference releases 21 to 24.^(34)^ Alpha-tocopherol values for several dark green leafy vegetables were extracted from a congress poster by Yang *et al.*^(35)^ We did not assess gamma-tocopherol intake because our food composition database did not include values for gamma-tocopherol. NutriSurvey software^(36)^ was used for data entry. No retention factors were used in the calculation of vitamin E intake, i.e. potential losses during cooking were not considered. Dietary supplements were not included in the analysis.

A background interview that included a range of questions, e.g. whether the girl was breastfeeding a child or was pregnant at the time of the study and whether she used tobacco, was conducted. Height was measured using a stadiometer and recorded to the nearest 0⋅1 cm. Weight was measured using a digital scale and recorded to the nearest 100 g. Body mass index (BMI)-for-age *z*-scores were calculated for non-pregnant girls using a SPSS macro by the World Health Organization.^(37)^

### Biological samples

Venous blood samples were taken from the antecubital vein. The samples were drawn using 21 G needles in plastic serum (10 ml) and K3EDTA (3 ml) tubes (BD Vacutainer^®^, Becton Dickinson International, Erembodegem, Belgium). The EDTA blood was centrifuged to plasma (Labnet Spectrafuge 6C, Labnet International, USA) at 4500 rpm (1700× g) for 10 min, and aliquots were stored in cryovials or microcentrifuge tubes. The serum tubes were allowed to stand at room temperature (22–36 °C) for 30 min and then centrifuged as described above. Biological samples were initially frozen and stored at −15 to −20 °C and transported to Maputo in a portable freezer by car. They were then shipped in dry ice to Finland, where they were stored at −70°C.

Plasma tocopherol concentrations were analysed at the National Institute for Health and Welfare, Finland (Laboratory accredited by Finas T077 (EN ISO-IEC 17025)). For tocopherol analyses, 0⋅8 ml of a 50 % ethanolic solution containing ascorbic acid, butylated hydroxytoluene (BHT), and tocol (internal standard for tocopherols) was added. After mixing, the analytes were extracted with 1 ml of hexane. An 0⋅8 ml hexane aliquot was evaporated under nitrogen, and the residue was dissolved in 120 μl of methanol. Gamma- and alpha-tocopherol were separated with an Inertsil ODS-3 column (2⋅1 × 100 mm, 3 μm, GL Sciences, Japan). The mobile phase was methanol 0⋅3 ml/min, 5 μl were injected into the column, and the tocopherols were detected by their fluorescence at 292/324 nm using peak height tocol ratios. Alpha-tocopherol was calibrated against reference standards. Vitamin E inadequacy was defined as plasma alpha-tocopherol below 12 μmol/l.^(9)^ An Architect ci8200 analyser (Abbott Diagnostics) was used for the enzymatic analysis of cholesterol (Abbott, 7D62–20).

Pregnancy was ascertained by a urine pregnancy test performed at the study centre or by the participant being visibly pregnant. If this information was not available, those who reported not being pregnant were coded as not pregnant and those who reported being pregnant were coded as missing. Information on the trimester of pregnancy was not available.

### Statistical methods

All of the descriptive analyses described below were done using post-stratification sampling weights based on the total population of 15- to 19-year-old girls in the five study areas given in the 2007 Census. They were done using the survey package in R version 4.0.4. We calculated the prevalence of vitamin E inadequacy. We used Mann–Whitey *U*-tests to compare energy and nutrient intake variables and tocopherol concentrations by season. For participants with more than one 24 h recall, the two first ones were used when statistically comparing the seasons. We determined the most important sources of vitamin E in the diets during the two seasons studied. This was done by calculating the mean daily intakes (as mg) and the proportion from different food groups using all 24 h recalls available for the sample included in this paper.

The analyses described below were done without sampling weights. We used linear multilevel regression analysis in SAS 9⋅4 (PROC MIXED covtest) to evaluate the associations between independent variables and plasma alpha-tocopherol. To take the clustering of participants in localities into account, we used two-level random intercept linear regression models. Locality (*n* 40) was treated as the higher-level unit and the individual girl as the lower-level unit.

Based on previous literature,^(6,10,20,38)^ we considered the following variables as potential predictors: age (years), BMI-for-age *z*-score, season, residence (rural/urban), pregnancy (yes/no), lactation (yes/no), malaria parasitaemia status (positive/negative), and human immunodeficiency virus (HIV) status (positive/negative). We did not include tobacco use since only 2 of the 504 girls with available questionnaire data responded that they used tobacco.

Five participants (1⋅0 %) had missing data for pregnancy and six (1⋅2 %) for lactation. Furthermore, two participants known to be non-pregnant had missing data for BMI-for-age *z*-score. Before regression analysis, these were imputed by hot deck imputation^(39)^ using area (five categories) as a deck variable. The numbers of missing values for malaria parasitaemia (17; 3 %) and for HIV (143; 28 %) were higher and were thus not imputed.

In Model 1 (*n* 508), we included age, season, rural–urban residence, pregnancy, and lactation as independent variables, but excluded the BMI-for-age *z*-score since it was not calculated for pregnant girls. In Model 2 (*n* 451), we excluded pregnant girls and added BMI-for-age *z*-score as a potential predictor, otherwise keeping the same variables as in Model 1. We also performed additional models to evaluate whether a positive malaria (Model 3, *n* 491) or HIV (Model 4, *n* 365) test was associated with plasma alpha-tocopherol. Models 3 and 4 included pregnant girls, and the other variables were the same as in Model 1. Finally, we performed Models 1–4 with a plasma alpha-tocopherol:total cholesterol ratio, gamma-tocopherol concentration, gamma-tocopherol:total cholesterol ratio, and gamma-tocopherol:alpha-tocopherol ratio as the dependent variable.

## Results

### Alpha-tocopherol intake

The median age of the sample at recruitment was 16 years. The participants were largely classified as having normal weight, and the majority lived in rural area (Table 1). The median reported energy intake was low at 5⋅15 MJ/d (Table 2). Median percentages of energy from fat and protein were also low, and carbohydrates dominated the diet. Alpha-tocopherol intake and density (mg/MJ) in the diet were higher in January–February than in May–June (Table 2). In January–February, when mangoes were in season, the main sources of alpha-tocopherol in the diet were mango and dark green leafy vegetables (Table 3). In May–June, the main sources were dark green leafy vegetables, groundnuts (groundnuts are harvested in April/May), and oil.

**Table 1.** Characteristics of the study sample (adolescent girls in Central Mozambique, *n* 508)

Variables	Data available, *n*	*N*	%
Age in years	508		
14–15		204	40⋅2
16–17		205	40⋅4
18–19		99	19⋅5
Urban/rural	508	161/347	31⋅7/68⋅3
Non-pregnant non-lactating	498	393	78⋅9
Pregnant	503	57	11⋅3
Lactating	502	51	10⋅2
Pregnant and lactating	498	3^a^	0⋅6
BMI-for-age *z*-score in the non-pregnant	444		
Thin or severely thin (<−2)		9	2⋅2
Normal weight (−2 to 1)		414	93⋅2
Overweight (>1)		21	4⋅7

a These participants are also included in the separate rows ‘pregnant’ and ‘lactating’.

**Table 2.** Energy and nutrient intake and tocopherol and cholesterol status in adolescent girls in Central Mozambique

	Total sample	January–February	May–June	Seasonal comparison, *P*-value^a^
**Dietary intake**				
*n* of participants	501	258^b^	243^c^	
Energy, MJ/d	5⋅15 (3⋅60–7⋅15)	4⋅78 (3⋅49–6⋅78)	5⋅59 (3⋅95–7⋅91)	0⋅006
Protein, % of energy	9⋅8 (7⋅9–12⋅1)	9⋅3 (7⋅3–11⋅6)	10⋅3 (8⋅6–12⋅7)	<0⋅001
Carbohydrate, % of energy	70⋅0 (62⋅0–76⋅4)	71⋅7 (65⋅2–77⋅0)	67⋅0 (59⋅7–75.0)	<0⋅001
Fat, % of energy	16⋅2 (10⋅5–23⋅7)	15⋅5 (9⋅7–20⋅6)	17⋅4 (11⋅3–25⋅3)	0⋅016
Saturated fatty acids, % of energy	6⋅4 (3⋅4–10⋅2)	6⋅3 (3⋅1–10⋅0)	6⋅7 (3⋅6–10⋅2)	0⋅63
Monounsaturated fatty acids, % of energy	3⋅8 (2⋅0–6⋅4)	3⋅3 (1⋅9–5⋅2)	4⋅5 (2⋅6–7⋅8)	<0⋅001
Polyunsaturated fatty acids, % of energy	3⋅0 (1⋅7–4⋅5)	2⋅7 (1⋅3–4⋅2)	3⋅3 (2⋅4–4⋅8)	<0⋅001
Alpha-tocopherol, mg/d	6⋅7 (4⋅0–11⋅2)	7⋅9 (5⋅0–13⋅0)	5⋅2 (3⋅0–8⋅6)	<0⋅001
Alpha-tocopherol density, mg/MJ	1⋅3 (0⋅7–2⋅1)	1⋅7 (1⋅1–2⋅4)	0⋅9 (0⋅5–1⋅6)	<0⋅001
**Plasma concentrations**				
*n* of participants	508	261	247	
Alpha-tocopherol, μmol/l	13⋅05 (11⋅05–15⋅80)	14⋅27 (12⋅29–16⋅39)	11⋅5 (9⋅73–13⋅49)	<0⋅001
Gamma-tocopherol, μmol/l	0⋅75 (0⋅49–1⋅01)	0⋅69 (0⋅45–0⋅96)	0⋅81 (0⋅57–1⋅10)	0⋅001
Gamma-tocopherol:alpha-tocopherol	0⋅06 (0⋅04–0⋅08)	0⋅05 (0⋅03–0⋅07)	0⋅07 (0⋅05–0⋅10)	<0⋅001
Total cholesterol, mmol/l	3⋅61 (3⋅00–4⋅11)	3⋅62 (3⋅02–4⋅07)	3⋅46 (2⋅94–4⋅16)	0⋅24
Alpha-tocopherol:total cholesterol, μmol/mmol	3⋅70 (3⋅20–4⋅23)	3⋅99 (3⋅49–4⋅55)	3⋅34 (2⋅96–3⋅79)	<0⋅001
Gamma-tocopherol:total cholesterol, μmol/mmol	0⋅21 (0⋅13–0⋅30)	0⋅19 (0⋅12–0⋅27)	0⋅23 (0⋅15–0⋅34)	<0⋅001

Medians and interquartile ranges.

a Mann–Whitney *U*-test.

b Intake calculated from one (*n* 154) or two (*n* 104) 24 h recalls per participant.

c Intake calculated from one 24 h recall per participant.

**Table 3.** Average daily intake of alpha-tocopherol from food groups and their contribution to the total intake among adolescent girls in Central Mozambique

	January–February, *n* 258^a^		May–June, *n* 335^b^	
Foods (main groups and selected sub-groups)	mg	%	mg	%
**Cereals**	**0⋅7**	**7**	**0⋅9**	**12**
Maize	0⋅6	6	0⋅8	12
**Roots and tubers**	**0⋅3**	**3**	**0⋅1**	**2**
**Vegetables**	**2⋅8**	**28**	**2⋅9**	**40**
Dark green leafy vegetables	2⋅7	27	2⋅7	38
**Fruit**	**4⋅7**	**48**	**0⋅3**	**5**
Mango	4⋅5	46	0⋅0	0
**Nuts and coconut**	**0⋅2**	**2**	**1⋅3**	**18**
Groundnuts	0⋅1	1	1⋅2	17
**Legumes**	**0⋅0**	**0**	**0⋅1**	**1**
**Meat, poultry, fish, and seafood**	**0⋅2**	**2**	**0⋅3**	**4**
**Oil**	**0⋅9**	**9**	**1⋅1**	**16**
**Other**	**0⋅2**	**2**	**0⋅2**	**2**
**Total alpha-tocopherol**	**9⋅8**	**100**	**7⋅2**	**100**

Values are weighted means and population proportions. The percentages of the main food groups may not add up to 100 due to rounding.

a Based on food consumption from one (*n* 154) or two (*n* 104) 24 h recalls per participant.

b Based on food consumption from one (*n* 255) or two (*n* 80) 24 h recalls per participant.

### Plasma concentrations

**Fig. 1.**
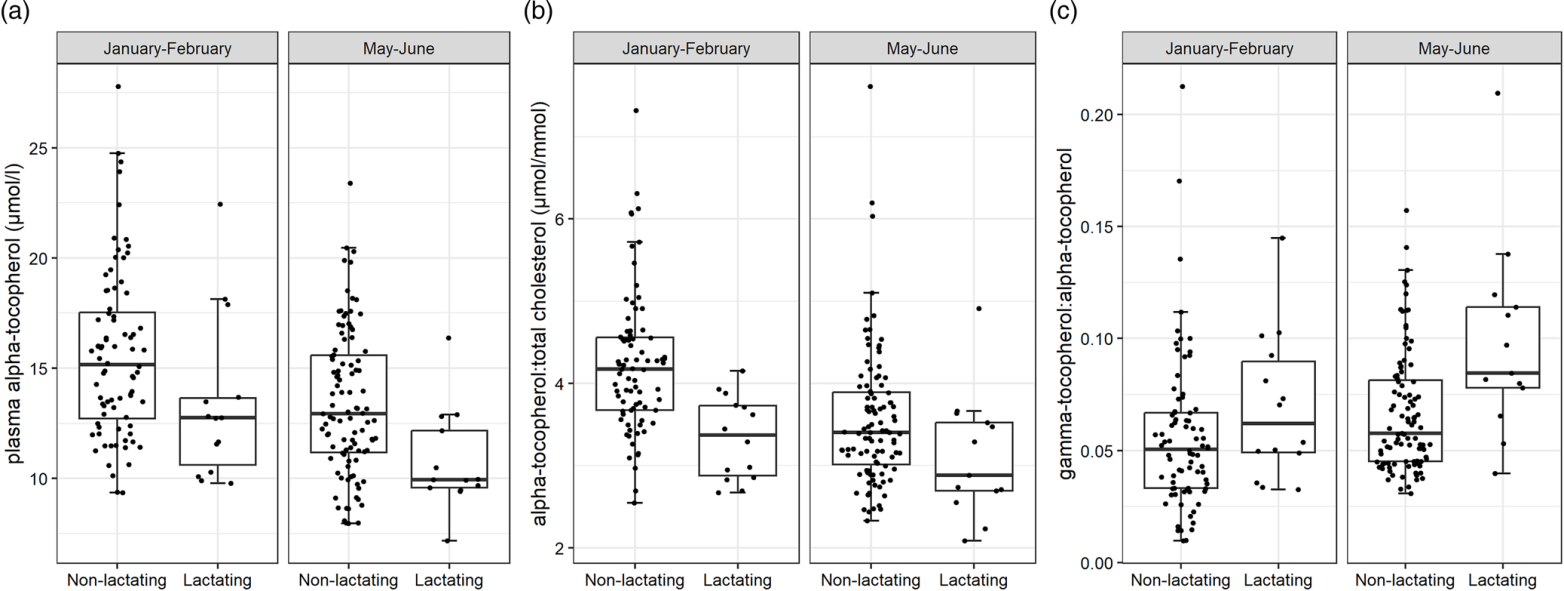
Box-plots illustrating the distributions of (a) plasma alpha-tocopherol concentrations, (b) alpha-tocopherol:total cholesterol ratios, and (c) gamma-tocopherol:alpha-tocopherol ratios in non-lactating and lactating adolescent Mozambican girls during two seasons. Each box represents, from top to bottom, the 75th percentile, median, and 25th percentile, and the whiskers show the values that fall within 1⋅5 interquartile ranges from each side of the box. Data points above the upper whiskers are outlying values. Data points were added over the box-plots with point scattering using geom_quasirandom in the ggbeeswarm package in R. Number of participants: 231 non-lactating and 26 lactating in January–February; 220 non-lactating and 25 lactating in May–June.

In the whole sample, the weighted mean plasma alpha- and gamma-tocopherol concentrations were 13⋅52 and 0⋅81 μmol/l, respectively. The weighted prevalence of vitamin E inadequacy was 36⋅7 % (95 % CI: 31⋅9–42⋅0). In non-pregnant non-lactating girls, the corresponding prevalence was 35⋅9 % (95 % CI: 30⋅3–42⋅0). Of the 51 lactating girls in this sample, 30 were classified as having vitamin E inadequacy (Table 4). The plasma alpha-tocopherol concentrations were higher (Table 2 and Fig. 1), and gamma-tocopherol concentrations were lower in January–February than in May–June (Table 2).

**Table 4.** Numbers and unweighted proportions of participants with vitamin E inadequacy (plasma alpha-tocopherol <12 μmol/l)

	Total sample			January–February			May–June		
Group	*n*	Vitamin E inadequacy, *n*	%	*n*	Vitamin E inadequacy, *n*	%	*n*	Vitamin E inadequacy, *n*	%
All participants	508	156	30⋅7	261	45	17⋅2	247	111	44⋅9
Non-pregnant non-lactating	393	114	29⋅0	198	28	14⋅1	195	86	44⋅1
Pregnant^a^	57	12	21⋅1	32	5	15⋅6	25	7	28⋅0
Lactating^a^	51	30	58⋅8	26	11	42⋅3	25	19	76⋅0

a Includes three participants who were pregnant and lactating.

### Predictors of tocopherol status

In the two-level regression analyses for all participants (Table 5), the season of the blood sampling, rural–urban residence, and lactation status were associated with plasma alpha-tocopherol concentration. When the dependent variable was changed to the other indicators of alpha-tocopherol status, i.e. alpha-tocopherol:total cholesterol ratio or the gamma-tocopherol:alpha-tocopherol ratio, the rural–urban residence was no longer a predictor.

**Table 5.** Linear two-level models (Model 1) for predictors of plasma tocopherol status indicators in adolescent Mozambican girls (*n* 508)

	Alpha-tocopherol, μmol/l		Alpha-tocopherol:total cholesterol ratio, μmol/mmol		Gamma-tocopherol:alpha-tocopherol ratio	
	Estimate (se)	*P*-value	Estimate (se)	*P*-value	Estimate (se)	*P*-value
**Fixed part**						
Constant	11⋅12 (1⋅84)		3⋅39 (0⋅45)		0⋅042 (0⋅017)	
Age, years	0⋅06 (0⋅11)	0⋅57	0⋅004 (0⋅028)	0⋅88	0⋅002 (0⋅001)	0⋅06
Season (January–February *v.* May–June)	2⋅20 (0⋅28)	<0⋅001	0⋅54 (0⋅07)	<0⋅001	−0⋅021 (0⋅003)	<0⋅001
Residence (urban *v.* rural)	3⋅10 (0⋅36)	<0⋅001	0⋅09 (0⋅12)	0⋅47	0⋅0005 (0⋅006)	0⋅93
Pregnant *v.* non-pregnant	0⋅56 (0⋅43)	0⋅19	0⋅06 (0⋅10)	0⋅54	−0⋅013 (0⋅004)	0⋅001
Lactating *v.* non-lactating	−1⋅98 (0⋅46)	<0⋅001	−0⋅65 (0⋅11)	<0⋅001	0⋅014 (0⋅004)	0⋅001
**Random part**						
Locality level	0⋅28 (0⋅23)		0⋅06 (0⋅02)		0⋅0002 (0⋅0001)	
Individual level	8⋅92 (0⋅58)		0⋅52 (0⋅03)		0⋅0007 (0⋅0000)	

	Gamma-tocopherol, μmol/l		Gamma-tocopherol:total cholesterol ratio, μmol/mmol	
	Estimate (se)	*P*-value	Estimate (se)	*P*-value
**Fixed part**				
Constant	0⋅38 (0⋅25)		0⋅143 (0⋅076)	
Age, years	0⋅03 (0⋅02)	0⋅039	0⋅007 (0⋅005)	0⋅12
Season (January–February *v.* May–June)	−0⋅16 (0⋅04)	<0⋅001	−0⋅045 (0⋅012)	<0⋅001
Residence (urban *v.* rural)	0⋅22 (0⋅08)	0⋅009	0⋅010 (0⋅025)	0⋅70
Pregnant *v.* non-pregnant	−0⋅15 (0⋅06)	0⋅009	−0⋅046 (0⋅018)	0⋅008
Lactating *v.* non-lactating	0⋅04 (0⋅06)	0⋅48	−0⋅003 (0⋅019)	0⋅88
**Random part**				
Locality level	0⋅03 (0⋅01)		0⋅003 (0⋅001)	
Individual level	0⋅16 (0⋅01)		0⋅014 (0⋅001)	

se, standard error.

Pregnancy was significantly associated with gamma-tocopherol concentration, gamma-tocopherol:total cholesterol ratio, and gamma-tocopherol:alpha-tocopherol ratio, but not with alpha-tocopherol or its ratio with cholesterol. In addition to pregnancy, in the two-level regression analyses for all participants (Table 5), the season of blood sampling and rural–urban residence were associated with plasma gamma-tocopherol concentration, but when the dependent variable was changed to gamma-tocopherol:total cholesterol ratio, rural–urban residence was no longer a predictor.

The results of the two-level regression analyses for non-pregnant participants (Table 6) were similar to those for all participants but additionally showed that a higher BMI-for-age *z*-score was associated with a lower alpha-tocopherol:total cholesterol ratio. Age was associated with gamma-tocopherol concentration but not with any other status indicator (Tables 5 and 6).

**Table 6.** Linear two-level models (Model 2) for predictors of plasma tocopherol status indicators in non-pregnant adolescent Mozambican girls (*n* 451)

	Alpha-tocopherol, μmol/l		Alpha-tocopherol:total cholesterol ratio, μmol/mmol		Gamma-tocopherol: alpha-tocopherol ratio	
	Estimate (se)	*P*-value	Estimate (se)	*P*-value	Estimate (se)	*P*-value
**Fixed part**						
Constant	10⋅86 (1⋅93)		3⋅67 (0⋅47)		0⋅042 (0⋅019)	
Age, years	0⋅08 (0⋅12)	0⋅51	−0⋅02 (0⋅03)	0⋅56	0⋅002 (0⋅001)	0⋅09
Season (January–February *v.* May–June)	2⋅35 (0⋅29)	<0⋅001	0⋅57 (0⋅07)	<0⋅001	−0⋅021 (0⋅003)	<0⋅001
Residence (urban *v.* rural)	3⋅09 (0⋅36)	<0⋅001	0⋅11 (0⋅12)	0⋅38	0⋅0003 (0⋅0061)	0⋅96
Lactating *v.* non-lactating	−1⋅99 (0⋅47)	<0⋅001	−0⋅68 (0⋅11)	<0⋅001	0⋅014 (0⋅004)	0⋅002
BMI-for-age *z*-score	0⋅13 (0⋅17)	0⋅44	−0⋅15 (0⋅04)	<0⋅001	−0⋅001 (0⋅002)	0⋅41
**Random part**						
Locality level	0⋅21 (0⋅24)		0⋅05 (0⋅02)		0⋅0002 (0⋅0001)	
Individual level	8⋅67 (0⋅60)		0⋅49 (0⋅03)		0⋅0008 (0⋅0001)	

	Gamma-tocopherol, μmol/l		Gamma-tocopherol:total cholesterol ratio, μmol/mmol	
	Estimate (se)	*P*-value	Estimate (se)	*P*-value
**Fixed part**				
Constant	0⋅349 (0⋅273)		0⋅147 (0⋅082)	
Age, years	0⋅034 (0⋅017)	0⋅046	0⋅001 (0⋅005)	0⋅19
Season (January–February *v.* May–June)	−0⋅142 (0⋅041)	0⋅001	−0⋅041 (0⋅012)	< 0⋅001
Residence (urban *v.* rural)	0⋅211 (0⋅084)	0⋅019	0⋅010 (0⋅027)	0⋅71
Lactating *v.* non-lactating	0⋅039 (0⋅065)	0⋅55	−0⋅008 (0⋅019)	0⋅69
BMI-for-age *z*-score	−0⋅007 (0⋅025)	0⋅78	−0⋅014 (0⋅007)	0⋅05
**Random part**				
Locality level	0⋅035 (0⋅013)		0⋅004 (0⋅001)	
Individual level	0⋅159 (0⋅011)		0⋅014 (0⋅001)	

se, standard error.

We found no association between positive malaria parasitaemia and any of the status indicators (results for Model 3 are not shown). HIV-positive status was associated with a higher gamma-tocopherol concentration (estimate 0⋅274, se 0⋅086, *P* = 0⋅002), gamma-tocopherol:total cholesterol ratio (estimate 0⋅106, se 0⋅026, *P*-value < 0⋅001), and gamma-tocopherol:alpha-tocopherol ratio (estimate 0⋅021, se 0⋅006, *P*-value < 0⋅001), but not with alpha-tocopherol concentration or alpha-tocopherol:total cholesterol ratio. Fig. 1 illustrates the alpha-tocopherol status indicators according to the season and lactation status.

## Discussion

Vitamin E inadequacy was highly prevalent among adolescent girls in Central Mozambique. We found a clear seasonal difference in alpha-tocopherol intake, which was also reflected in plasma alpha-tocopherol concentrations. Having the blood sample taken during the mango season (January–February) was associated with a higher plasma alpha-tocopherol concentration and a lower gamma-tocopherol concentration. Being a lactating mother was associated with poorer alpha-tocopherol status. Season and lactation were consistently associated with alpha-tocopherol status regardless of which the three status indicators were used as the dependent variable. Season and pregnancy were consistently associated with gamma-tocopherol status, regardless of whether its concentration or ratio to cholesterol was used as the dependent variable.

Regardless of the season, girls living in rural area were more vulnerable to having low plasma alpha-tocopherol concentrations. However, the significance of rural–urban residence as a predictor disappeared when the alpha-tocopherol:total cholesterol ratio or the gamma-tocopherol:alpha-tocopherol ratio was used as a status indicator. This can be explained by the fact that while the urban girls tended to have higher alpha-tocopherol concentrations, they also tended to have higher total cholesterol concentrations^(40)^ and higher gamma-tocopherol concentrations than their rural peers.

A higher BMI-for-age *z*-score was associated with a lower alpha-tocopherol:total cholesterol ratio because girls with higher BMI-for-age *z*-score also tended to have higher total cholesterol (*P*-value < 0⋅001, variables as in Model 2, data not shown). Being HIV-positive was associated with a higher gamma-tocopherol:alpha-tocopherol ratio because although HIV was not associated with alpha-tocopherol, HIV-positive girls had higher gamma-tocopherol concentrations than their HIV-negative peers. This is in line with findings in HIV-positive adolescents in the USA.^(41)^ Finally, being pregnant was associated with a lower gamma-tocopherol:alpha-tocopherol ratio because pregnant girls had lower gamma-tocopherol concentrations than their non-pregnant peers, a finding that is in accord with the study in Gambia.^(20)^

The plasma concentration of alpha-tocopherol found in this study (mean 13⋅52 μmol/l, median 13⋅05 μmol/l) was lower than that typical for high-income settings^(11)^ but was roughly similar to reports from neighbouring countries. In previous studies, 10- to 19-year-old girls in Malawi had a mean serum concentration of alpha-tocopherol of 10⋅2 μmol/l,^(14)^ 5- to 10-year-old children in Tanzania had a median of 15⋅4 μmol/l,^(16)^ and pregnant women in Tanzania had a mean of 15⋅4 μmol/l.^(24)^ In contrast, a study among the elderly in an informal settlement in South Africa found a strikingly low mean concentration of 4⋅8 μmol/l in women. The diet of these elderly participants was described as monotonous, high in maize meal and bread, and low in micronutrient-rich foods.^(18)^ The median gamma-tocopherol:alpha-tocopherol ratio found in our study population was similar to that reported in pregnant women in Bangladesh,^(3)^ and the median plasma concentrations of gamma-tocopherol were similar to those found in children in Tanzania^(16)^ and pregnant women in Tanzania^(24)^ and Nigeria.^(25)^

In high-income settings, the consumption of oils and other fats is typically high and can provide up to 40 % of the total alpha-tocopherol in the diet.^(7,42)^ Comparisons with other Sub-Saharan African populations cannot be done because reports on the sources of dietary alpha-tocopherol are lacking. Relative to high-income settings, the intakes of oils and other fats and their importance as a source of alpha-tocopherol were very low in our sample. In this low-fat diet, several other food groups, such as mangoes, dark green leafy vegetables, and groundnuts, clearly had a more important role in the supply of alpha-tocopherol than in high-income settings. Food composition databases, including ours, often do not contain values for gamma-tocopherol, making it difficult to assess its intake and dietary sources. Heinonen and Piironen^(43)^ reported that the main sources of gamma-tocopherol in Finnish diets were fats, such as vegetable oils and margarines, but similar reports from Sub-Saharan Africa are not available.

Recent papers from Mozambique have illustrated how unhealthy ultra-processed foods and drinks have made their way into the food environment as part of the process of nutrition transition,^(44,45)^ and urbanization is predicted to have negative effects on diet quality.^(46)^ Although overweight was still rare in our data, it has been reported that overweight and obesity are on the rise in Mozambique, particularly in women.^(47)^ Thus, there is an urgent need to design and implement public health policies and initiatives that aim to prevent the increasing adoption of ultra-processed foods and drinks in the diet^(48)^ and to simultaneously encourage the consumption of traditional foods, such as dark green leafy vegetables, fruit, nuts, and seeds, which are rich in alpha-tocopherol and other micronutrients. These foods play a vital role in maintaining a normal weight and preventing chronic diseases.^(49)^ This is true in both rural and urban areas. In addition, the poorer alpha-tocopherol status in rural Mozambique suggests that dietary diversification would specifically benefit the rural poor. While better diets are the preferred long-term approach for enhancing micronutrient intakes, vulnerable groups, such as pregnant women, would also benefit from multiple micronutrient supplementation.^(50)^

Our findings show that lactating adolescent mothers may comprise a specific risk group for vitamin E deficiency in resource-poor settings. Lactating mothers secrete alpha-tocopherol into breast milk, and the highest concentrations in breast milk are found in colostrum.^(51)^ At a later point in the lactation period, alpha-tocopherol concentrations between 3⋅5 and 5⋅7 mg/l have been detected in the breast milk of non-vitamin E-supplemented European women^(6)^. In Tanzania, a study in HIV-positive women found similar concentrations in breast milk (4⋅4 and 3⋅8 mg/l at 3 and 6 months post-partum, respectively) in a non-vitamin E-supplemented group.^(52)^ Based on data from European women, the EFSA has estimated that the secretion of alpha-tocopherol during exclusive breastfeeding can be assumed to be approximately 4 mg/d.^(6)^ In light of this estimate of additional loss due to lactation, our results suggest that lactating adolescent mothers in Mozambique did not have sufficiently high dietary intake or body stores to maintain alpha-tocopherol status similar to that of their non-lactating peers. This finding is in line with those of two prospective studies in lactating women. Papathakis *et al.*^(23)^ found a clear decrease in plasma alpha-tocopherol concentrations from 6 to 24 weeks post-partum in HIV-negative and HIV-positive groups of South African women. Similarly, da Silva *et al.*^(53)^ noted that serum alpha-tocopherol concentrations decreased during the lactation period in a group of Brazilian women. The dietary intake of vitamin E among these Brazilian women was estimated to be in a roughly similar range to that in our sample of Mozambican girls. Interestingly, da Silva *et al.* also found that the breast milk concentrations of alpha-tocopherol did not decrease during the same time period (first measurement at approximately 1– 2⋅5 months post-partum and the last measurement at around 3–4⋅5 post-partum), which supports the idea that the infant's supply of this nutrient is prioritized over the status of the mother.

The strengths of this study include the analysis of two different seasons and a sample of participants from both urban and rural areas. However, as we have discussed previously,^(54)^ a limitation of our study is that the low reported daily energy intakes are likely to be a result of underreporting during the 24 h recall interviews, which in turn may have affected our estimates of alpha-tocopherol intake. Very few studies in Sub-Saharan Africa have used biomarkers to assess the validity of dietary assessment methods,^(55)^ and more such studies are needed to enhance our understanding of the accuracy with which intakes can be captured in different settings. Another limitation is that we did not have data on the post-partum stage of the lactating girls at the time of the study. Such data, especially had the number of lactating participants been larger, might have provided further insights into plasma values according to the lactation stage. Moreover, when examining the association between HIV status and plasma alpha-tocopherol concentrations, a large number of participants had to be excluded due to missing data, which may have caused bias in the analysis.

## Conclusions

About one-third of adolescent girls in our sample from Central Mozambique were classified as having inadequate vitamin E status. Mangoes were an important source of vitamin E when they were available, but especially outside the mango season, there is a need to improve vitamin E intakes. We suggest that lactating adolescent mothers are a risk group for poor vitamin E status in resource-poor settings.
